# Effects of Money on Utilitarian and Deontological Inclinations in Moral Judgments: A Study Using Process-Dissociation Approach

**DOI:** 10.3390/bs15040430

**Published:** 2025-03-27

**Authors:** Mufan Zheng, Huijun Li, Liqinzi Mo, Xiaoxiao Wang

**Affiliations:** 1 Department of Psychology, Wuhan University, Wuhan 430072, China; zhengmufan@whu.edu.cn (M.Z.); 2017301270014@whu.edu.cn (L.M.); 2 School of Law, South-Central Minzu University, Wuhan 430074, China

**Keywords:** money, love of money, moral judgments, deontology, utilitarianism, process-dissociation approach

## Abstract

Previous research has extensively examined the impact of money on morality, yet limited attention has been given to how the mere contemplation of money influences moral reasoning and decision-making. The present study aims to address this gap by exploring how both the concept of money and the love of money shape deontological and utilitarian inclinations in moral judgments. In Study 1 (*N* = 102), we investigated the effect of money concept priming on moral thinking. Participants were primed with either the concept of money or a neutral concept through a scrambled-sentences task, and subsequently made moral decisions in 20 dilemmas adapted from Conway and Gawronski. These dilemmas required participants to decide whether to harm others in order to achieve a greater outcome. To assess participants’ utilitarian and deontological tendencies, we employed the process-dissociation procedure. In Study 2 (*N* = 488), we further examined the relationship between the love of money—a long-term trait—and moral judgments. Specifically, we investigated whether four moral orientations (deliberation, rule, sentiment, and integration) mediate the relationship between the love of money and deontological/utilitarian inclinations. Participants completed the love of money scale and the moral orientation scale before reading the same 20 dilemmas from Study 1. Our findings revealed that priming the concept of money enhanced utilitarian tendencies but did not significantly affect deontological tendencies. Furthermore, the love of money was negatively correlated with deontological tendencies and positively correlated with utilitarian tendencies. Deliberation fully mediated the effect of the love of money on utilitarian tendencies and partially mediated its effect on deontological tendencies.

## 1. Introduction

Money, as a pervasive element in contemporary life, exerts a profound influence on individuals’ cognitive processes and behaviors (Zaleskiewicz et al., 2018). A substantial body of research has examined the relationship between money and morality. Some scholars have argued that the mere presence of money can lead to a decline in moral standards (Tang et al., 2018; Tong et al., 2013) and a reduction in prosocial behaviors (Gasiorowska et al., 2016; Kouchaki et al., 2013; Mead & Stuppy, 2014). Conversely, other researchers have pointed to a positive correlation between currency, trade, and markets with prosocial behavior, trust, universal moral values, and moral emotions (Enke, 2023; Henrich et al., 2010). However, relatively few empirical studies have explored how money-related cognition shapes moral reasoning in the context of ethical dilemmas.

The influence of money-related thinking on ethical decision-making is especially pronounced when individuals face choices involving trade-offs between costs and benefits. Whether individuals activate a money-related mindset or possess a long-term orientation toward money can significantly shape their decisions. For example, environmental protection has become a prominent issue in recent years. Governments must often navigate choices between economic development and environmental conservation, while business leaders are tasked with balancing—or even deciding between—increasing profits and fulfilling social responsibilities. We hypothesize that government officials who prioritize taxation in their decision-making may make significantly different choices than those who do not. Similarly, business owners focused on accumulating wealth may approach decisions differently than those with a stronger commitment to social responsibility. In light of these considerations, the present study aims to examine how money influences moral judgments, particularly in contexts where cost–benefit trade-offs are prominent.

### 1.1. Moral Judgment and Process-Dissociation Approach

#### 1.1.1. Moral Judgment

Moral judgment has been defined as the evaluation of a person’s actions or character as good or bad, based on a set of virtues deemed obligatory by a particular culture or subculture (Haidt, 2001). Typically, individuals assess actions according to two primary ethical principles: utilitarianism and deontology. Deontological judgments emphasize adherence to established rules, principles, and duties, asserting that certain actions are intrinsically right or wrong, irrespective of their consequences. In contrast, utilitarianism seeks to maximize overall happiness or well-being for the greatest number of people, focusing on the outcomes of actions (Gawronski & Beer, 2017; Gawronski et al., 2017). Traditionally, moral judgments were conceptualized as the result of logical reasoning, as exemplified by Kohlberg’s (1969) stage theory of moral development, which posited that the moral principles guiding individual reasoning are contingent on one’s developmental stage.

However, this framework was challenged by Haidt (2001), who introduced a novel perspective suggesting that moral judgments are fundamentally intuitive. According to Haidt, moral decisions can be made automatically and effortlessly, without the need for deliberate contemplation. A prominent example of this intuitive process is the phenomenon of moral dumbfounding, in which individuals find themselves unable to justify their moral choices. This inability arises from reliance on intuitive moral responses, which often bypass explicit reasoning.

Building on Haidt’s foundational work, Greene et al. developed the dual-process theory of moral judgment (Greene et al., 2001, 2004, 2008; Greene, 2009). This theory is primarily grounded in classical sacrificial dilemmas, such as the footbridge and trolley problems, where participants must decide whether to sacrifice a few lives to save many. Greene’s model suggests that the act of harming others in these scenarios activates intuitive and emotional responses, which predispose individuals toward deontological judgments. These judgments are marked by a reluctance to inflict harm, even if doing so would maximize overall welfare. In contrast, when moral intuition or emotion is not engaged, or when individuals lack the propensity for intuitive moral processing, an alternative cognitive pathway is activated. In these cases, individuals engage in rational, deliberate, and effortful reasoning, which is more likely to lead to utilitarian judgments that prioritize the greatest good for the greatest number.

#### 1.1.2. Process-Dissociation Procedure

Traditionally, utilitarian and deontological inclinations have been conceptualized as opposite ends of a bipolar continuum. However, Conway and Gawronski (2012) challenged this view by proposing that utilitarian and deontological inclinations are conceptually distinct and functionally independent processes. They argued that ethical judgments can be simultaneously influenced by both types of inclinations, rather than being confined to one or the other. Furthermore, they observed that classical moral dilemmas, such as the footbridge dilemma, are insufficient for analyzing the specific contributions of utilitarian and deontological inclinations in moral decision-making. To address this limitation, they adapted the Process-Dissociation (PD) approach, originally developed by Jacoby (1991), to examine the interplay between utilitarian and deontological tendencies in moral reasoning.

Consistent with Conway and Gawronski’s framework, the present study employs the Process-Dissociation procedure to investigate how exposure to money influences utilitarian and deontological tendencies in moral judgments. While most dilemmas used in the PD approach do not explicitly involve economic themes, all such dilemmas require decision-makers to weigh benefits against costs. For instance, dilemmas may include decisions such as whether to suffocate a baby to prevent enemy forces from discovering and killing all villagers, whether a father should allow his daughter to star in a pornographic film to prevent his family from starving, or whether a doctor should use a vaccine to control a large-scale, potentially deadly epidemic, despite its side effects that might result in some deaths. In each of these scenarios, decision-makers must assess what must be sacrificed and what benefits can be gained, even if these benefits are not economic but rather related to the greater good of all involved. This mode of decision-making can be profoundly influenced by the concept of money and the long-term pursuit of wealth.

### 1.2. Money

#### 1.2.1. Theories of Money in Anthropology and Sociology

Anthropology reconceptualizes money beyond its economic functions, viewing it as a cultural artifact that encodes meanings, mediating social hierarchies, ritual obligations, and identity (Nelms & Maurer, 2014). This semiotic perspective aligns with psychological evidence showing that mere exposure to money can trigger self-serving cognitions (Bijleveld & Aarts, 2014), while the act of spending or receiving money can modulate affective states and intrinsic motivations.

Money has long been a focal point of anthropological inquiry, with its multifaceted nature and pervasive influence on human societies making it a compelling subject for scholarly examination. One key insight emerging from anthropological research is the recognition that material currencies—such as shells, coins, and banknotes—serve functions extending beyond their conventional roles as mediums of exchange and stores of value (Nelms & Maurer, 2014). This observation underscores the complexity of money, highlighting its capacity to acquire and convey meanings that are not solely economic in nature.

This anthropological framework resonates with the psychological tradition of studying money through the lens of semiotic processes, which involve the construction of meaning in human interactions with money. This approach has revealed how money can evoke psychological responses and shape behavior, often with unintended consequences. For example, receiving money can influence intrinsic motivation, altering the degree to which individuals derive pleasure from a given task. Similarly, spending money can elicit predictable shifts in affective states, reflecting the emotional significance of financial transactions. Moreover, mere exposure to money, even in the absence of direct transactions, has been shown to trigger self-serving behaviors and cognitions, indicating that money’s symbolic power extends well beyond its material utility (Bijleveld & Aarts, 2014).

Scholars have diverging perspectives on the role and meaning of money. One influential theory examines money from a functional standpoint, viewing it as a medium of exchange, a unit of account, a store of value, and a standard of deferred payment. Traced back to Aristotle, this perspective argues that money resolves the “double coincidence of wants” inherent in barter economies (Jevons, 1875), facilitating the exchange and valuation of diverse goods and thereby streamlining economic transactions (Nelms & Maurer, 2014). Another major viewpoint emphasizes the role of social relations and conventions in the creation of money, focusing on trust among market participants and the state’s authority in enforcing contracts denominated in national currency. This perspective highlights the interplay between economic practices and social institutions in shaping the meaning and function of money (Innes, 1913, 1914; Knapp, 1905/1924).

Marx, Weber, and Simmel each developed theoretical frameworks on money within their classic sociological works. Money, according to these theorists, serves as a universal and internally homogeneous measure that can “commensurate incommensurabilities” (Carruthers & Espeland, 1998, p. 1400), eliminating qualitative differences by adopting a singular numerical scale. It enforces impersonal, rational, instrumental, and calculative modes of thinking, alienates individuals from the material world, and weakens social relations, thus fostering individualism (Gilbert, 2005, p. 379). Simmel (1950, p. 412) famously characterized money as “transforming the world into an arithmetic problem”, linking its use to psychological shifts that prioritize formalized, quantitative calculations for personal gain. This narrative also reinforces the assumption that numeracy and quantification are central to modern life.

For Marx, commodity money is a central force in regulating capitalist production and exchange. Marx referred to money as a “privileged commodity”, which, while a commodity itself, stands apart for its ability to serve as a general measure of the exchange value of other commodities (Marx, 1976, p. 187). He argued that this “riddle of the commodity fetish”, once made visible, reveals how money obscures the social relations underlying capitalist production (Marx, 1976, p. 187). Similarly, Weber and Simmel saw money as central to the social and economic transformations of the 19th and early 20th centuries. Weber highlighted the role of the state in the creation of money and the regulatory function of bureaucracy in its circulation, stressing that money facilitates “monetary calculation”, enabling the comparison and valuation of goods and services (Weber, 1978, p. 81). In this way, Weber viewed money as part of the rationalization of modern life, where expression in monetary terms yields the highest degree of formal calculability (Weber, 1978, p. 85). Simmel’s (1907/2004) approach, while also emphasizing the social change driven by money, argued that its emergence as a universal equivalent had both liberating and homogenizing effects. In Simmel’s view, money’s ability to free individuals from hereditary status paradoxically led to a form of egalitarianism that erased established hierarchies, positioning money as a central instrument in regulating social relations (Nelms & Maurer, 2014).

#### 1.2.2. Tool Theory and Drug Theory

Some anthropologists have argued that the use of money implies a psychological predisposition, specifically the mental state of the calculating “economic man” (Malinowski, 1984/1922, p. 173). Neurobiological processes further validate two distinct categories that shape individuals’ relationship with money: “tool” and “drug” (Lea & Webley, 2006, 2014). From the tool perspective, money is seen as a means to achieve goals, whereas the drug theory explains instances in which money becomes a “non-functional incentive”, mimicking biological rewards and continuing to influence behavior in a delusional and non-functional manner (Bijleveld & Aarts, 2014).

Tool theory encompasses all the ways in which money satisfies needs and desires rooted in biological instincts. Despite its multiple functions (e.g., as a store of value, medium of exchange, and unit of account), money essentially serves to help individuals obtain what they need and desire. Lea and Webley (2014) have used the drug theory to describe these non-tool aspects of money-related behavior. Metaphorically, the drug theory posits that money operates like a biological drug, influencing behavior in ways that are neither adaptive nor tool-based, yet still engaging reward-related mechanisms in the human brain that have evolved over time. Money, in this view, provides a biological incentive, guiding attention and eliciting goal-directed behavior without conscious awareness (Bijleveld & Aarts, 2014).

#### 1.2.3. Empirical Research About Money and Behaviors

Empirical research has aligned with these theoretical perspectives, revealing that money exerts both positive and negative influences on individual psychology. A series of studies have shown that individuals primed with money tend to prefer completing tasks independently, generally favor solitude, and spend less time helping others when given the opportunity to do so (Vohs et al., 2006, 2008). Money activates self-improvement goals that promote self-centered behaviors, such as purchasing goods for oneself, while simultaneously suppressing other-oriented goals that typically motivate and facilitate prosocial behaviors, such as helping others (Nelms & Maurer, 2014).

However, some studies have also demonstrated a positive relationship between money and prosocial behavior, based on the functional perspective of money. For example, a coding analysis of the cultural folklore of 943 pre-industrial ethno-linguistic groups found that the degree of market interaction in a society (primarily measured by trade between communities and the presence of currency) was significantly positively correlated with prosocial behavior, interpersonal trust, universal moral values, and moral emotions such as guilt, shame, and anger. The authors argued that morality is functional, evolving to support human cooperation. Consequently, the existence of markets may encourage prosocial behavior, as cooperation within markets benefits individuals (Enke, 2023). Another study found that using money to help others or engaging in altruistic spending led to more positive emotional experiences (Aknin et al., 2018).

Similarly, a study involving three behavioral experiments across 15 different populations found that market integration (measured by the percentage of calories purchased) was positively correlated with fairness, while community size was positively correlated with punishment. The researchers argued that evolutionary mechanisms related to kinship and reciprocity form the foundation of primate sociality but do not easily extend to large, unrelated groups. They proposed that the evolution of larger societies required norms and institutions that maintain fairness in fleeting exchanges. If true, participation in larger-scale institutions such as markets and world religions should be associated with greater fairness, and larger communities should engage in more punishment of unfairness. Based on these findings, they concluded that modern prosociality reflects not only innate psychological mechanisms but also the norms and institutions that have emerged throughout human history (Henrich et al., 2010).

#### 1.2.4. Summary

In summary, scholars across disciplines consistently conceptualize the influence of money on human societies and individuals through a dualistic lens, recognizing its capacity to generate both beneficial and detrimental consequences. Anthropological and sociological frameworks (e.g., Marx, 1976; Simmel, 1907/2004; Nelms & Maurer, 2014) converge in conceptualizing money as a cultural technology that reconfigures social relations—enabling market rationalization (Weber, 1978), alienating qualitative experiences into quantifiable metrics (Carruthers & Espeland, 1998), and serving as a “privileged commodity” in capitalist systems. This dialectical tension is mirrored in psychological theories that position money as a dual-natured entity: Neurobiological models distinguish its “tool” functions from its “drug-like” capacity to hijack reward pathways (Lea & Webley, 2006; Bijleveld & Aarts, 2014), while behavioral evidence demonstrates its paradoxical effects—reinforcing both self-serving individualism (Vohs et al., 2006) and institutionalized prosociality (Henrich et al., 2010; Enke, 2023).

The current study adopts this dualistic framework to examine the impact of money on moral cognition. We posit that monetary cognition fosters a market-oriented mindset—a mental schema that paradoxically reconfigures ethical decision-making. On the one hand, this schema may encourage interpersonal indifference and normative disengagement through its emphasis on transactional logic (e.g., deprioritizing communal obligations). On the other hand, it may incentivize institutional-level optimization of collective welfare by prioritizing systemic efficiency over particularistic moral imperatives. This cognitive shift may concurrently intensify utilitarian reasoning (e.g., cost–benefit analysis of aggregate outcomes) while attenuating deontological deliberation rooted in categorical duties.

### 1.3. Money and Moral Judgments

Utilitarianism and deontology are two distinct moral theories that individuals may invoke when reasoning through moral dilemmas. However, it is important to note that utilitarianism does not necessarily equate to a decline in moral standards; rather, it represents a distinct mode of thinking, one that contrasts with deontological ethics. In this regard, our research diverges from prior studies that have focused on how money influences moral standards. Instead, we aim to explore the impact of money on the styles of moral reasoning and the cognitive shifts associated with these processes. This focus sets our investigation apart from previous work in this domain.

Zaleskiewicz et al. (2020) examined the relationship between a market mindset and moral judgments, finding that exposure to market relationships increases individuals’ tendency to make utilitarian moral choices. While market mindset and thoughts of money share similar connotations, they represent distinct theoretical constructs. A market mindset is activated when individuals engage in market transactions, promoting proportional thinking and a focus on investments and returns. In contrast, money, as the most pervasive medium of exchange, extends beyond the scope of market transactions and permeates various aspects of daily life (Jiang et al., 2014). Although thinking about money may evoke a mindset similar to that of engaging in market transactions (Kouchaki et al., 2013; Tong et al., 2013), different aspects of money can trigger diverse psychological processes and behaviors. For instance, individuals may perceive, save, spend, lack, or give away money, each of which can provoke distinct cognitive and emotional responses (Jiang et al., 2014). Moreover, Zaleskiewicz et al. (2020) employed classical moral dilemmas to assess individuals’ moral judgments, yet this approach fails to isolate the specific contributions of deontological and utilitarian inclinations during moral reasoning. Thus, the present study aims to investigate the relationship between money and deontological versus utilitarian moral thinking using the process-dissociation technique.

The dual-process model of moral judgment (Greene, 2009; Greene et al., 2001) posits that individuals’ reliance on either deontological or utilitarian principles during moral decision-making is influenced by two distinct psychological processes: automatic, intuitive, and affective processing, as well as deliberate, effortful, and cognitive processing. Research on sacrificial moral dilemmas has shown that when individuals are confronted with situations requiring them to harm others to save a larger number, they often experience strong emotional reactions and moral intuitions. These reactions lead them to intuitively reject harm, favoring deontological choices, even when the alternative would result in a greater overall benefit. Conversely, in less emotionally charged situations, individuals engage in more cognitive processing, opting to sacrifice the few to save the many. Studies have consistently found that utilitarian moral reasoning typically relies on cognitive, effortful processing, whereas deontological thinking is more often based on intuitive and automatic responses (Bartels, 2008; Greene et al., 2008; Hayakawa et al., 2017; Koenigs et al., 2007; Kroneisen & Steghaus, 2021; Li et al., 2018; Moore et al., 2008; Patil et al., 2020).

Recent scholarship has also proposed that additional cognitive orientations, such as rule orientation and deliberation orientation, may influence moral judgment. However, these orientations have not yet been fully integrated or tested within the dual-process framework. Rule orientation is associated with stronger deontological inclinations and weaker utilitarian inclinations, while deliberation orientation is linked to increased deontological tendencies and decreased utilitarian tendencies (Fleischmann et al., 2019).

As discussed in prior theories and research on money, we hypothesize that money’s influence on individuals encompasses both positive and negative aspects. On the negative side, money can engender phenomena such as the “commodity fetish” described by Marx (1976) and the idea of “transforming the world into an arithmetic problem” articulated by Simmel (1950). These perspectives suggest that money may narrow one’s focus to personal gain, foster indifference toward others and society, and promote a selfish, individualistic mindset. Such a shift can erode social cohesion and diminish moral standards (Jiang et al., 2014; Kouchaki et al., 2013; Mead & Stuppy, 2014). It may also lead individuals to engage in cost–benefit analyses that prioritize self-interest, neglecting fundamental moral imperatives such as caring for others and avoiding harm (Falk & Szech, 2013; Gasiorowska et al., 2016; Kraus et al., 2010; Reniers et al., 2012; Wang & Krumhuber, 2017), thereby favoring utilitarian decisions and diminishing deontological inclinations.

On the other hand, money can also exert positive influences. For instance, market-oriented thinking and the ubiquitous nature of currency can foster a mindset geared toward cooperation and mutual benefit (Enke, 2023; Henrich et al., 2010). This orientation may lead to more utilitarian decision-making, which aims to maximize collective welfare. Moreover, money can have neutral cognitive effects by promoting a calculative mindset that emphasizes economic rationality (Liu & Aaker, 2008). This inclination toward economic calculation may reinforce utilitarian tendencies, as decisions are increasingly based on cost–benefit evaluations within an economic framework.

Numerous studies have examined the effects of money priming on psychological processes and behavior (Vohs, 2015). However, money is a multifaceted construct, encompassing various aspects such as money as a concept (Liu & Aaker, 2008), counting money (Zhou et al., 2009), desire for money (Tang et al., 2018), and income level (Côté et al., 2013). These facets are distinct and may have differential impacts on behavior. The present study focuses on how the concept of money and the desire for money influence moral judgments. Specifically, the desire for money refers to individuals’ perceptions of the meaning of money, their attitudes toward it, and their desires to acquire it (Tang et al., 2018; Tang & Chiu, 2003).

We hypothesize that the mere activation of money-related thoughts and the long-term trait of loving money may exert differential effects on individuals. Priming money-related concepts may primarily induce a cost–benefit analysis mindset in participants, aligning with the tool theory perspective (Lea & Webley, 2014). This cognitive orientation is likely to be more closely associated with utilitarian decision-making. In contrast, the love of money, as a long-term individual trait, may have a deeper and more pervasive impact on one’s values and cognitive processes. This trait may align more with the drug theory, wherein the influence of money acts as a biological incentive (Lea & Webley, 2014). Individuals who possess this trait may not only develop a heightened calculative mindset but may also be more prone to selfish behaviors, self-serving biases, and general indifference toward others (Gasiorowska et al., 2016; Mead & Stuppy, 2014; Tang et al., 2018; Wang & Krumhuber, 2017). Nevertheless, the desire for money may also cultivate a more business- or market-oriented cognitive framework, encouraging individuals to approach social interactions and decision-making from a cooperative, win–win perspective. While this mindset may foster utilitarian inclinations, it may represent a more adaptive form of utilitarianism, characterized by a focus on maximizing collective welfare rather than personal gain. Therefore, we hypothesize that the love of money will correlate with increased utilitarian tendencies and decreased deontological tendencies.

When individuals encounter money-related situations, they often activate a business-oriented decision-making mindset, concentrating on maximizing and quantifying utility and eliciting a cost–benefit analysis (Liu & Aaker, 2008). Based on the dual-process model, this cognitive evaluation process is typically associated with increased utilitarian moral thinking and decreased deontological thinking. We hypothesize that deliberation orientation will mediate the effect of money on increased utilitarian moral judgments and decreased deontological judgments. Moreover, individuals with a high desire for money may be more likely to prioritize self-interest over moral standards, exhibiting reduced empathy and concern for others (Falk & Szech, 2013; Gasiorowska et al., 2016; Kouchaki et al., 2013; Kraus et al., 2010; Reniers et al., 2012; Wang & Krumhuber, 2017). These tendencies are often associated with decreased deontological thinking, and we expect that rule-oriented thinking will mediate the effect of money on reduced deontological moral judgments.

### 1.4. The Current Study

The present research aims to investigate the influence of money on moral judgments through two studies. Study 1 employs an experimental priming paradigm, priming the concept of money to examine its immediate impact on moral reasoning. Study 2 focuses on assessing participants’ long-term attitudes toward money. Across both studies, we utilize the Process-Dissociation (PD) procedure to evaluate participants’ inclinations toward deontological and utilitarian moral judgments.

In addition to examining the direct effects of money on moral judgments, Study 2 explores potential mediating factors. We assess four key moral orientations—deliberation, rule, sentiment, and integration—to determine whether these orientations mediate the relationship between money and moral thinking. Specifically, we hypothesize that priming the concept of money will enhance utilitarian inclinations, while the love of money will be associated with increased reliance on utilitarianism and reduced reliance on deontological principles. Furthermore, we expect that deliberation and rule orientations will significantly mediate the relationship between money and moral judgments.

## 2. Study 1

### 2.1. Method

#### 2.1.1. Participants and Design

We used G*Power to calculate the required sample size for the experiment, specifying a medium effect size (*d* = 0.5), an alpha level of 0.05, and a power of 1 − *β* = 0.8. The calculated sample size for the *t*-test was 102 participants. A total of 102 participants (37 male, 65 female; age range: 18–45 years, *M* = 22.53, *SD* = 5.72) were recruited via an online platform (www.wenjuan.com; accessed on 20 August 2023) in China. The sample included 84 students (82.4%) and 13 employed individuals (17.6%), with 52 participants (51%) from rural areas and 50 participants (49%) from urban areas. Participants were randomly assigned to either the money concept priming group or the neutral prime group. All participants volunteered for the study.

#### 2.1.2. Procedure and Materials

To prime the concept of money, participants first completed a scrambled-sentences task (Vohs, 2015), which consisted of 30 sets of five words each. Participants were instructed to form a coherent sentence using four out of the five words within a specified time limit. For the neutral-prime group, the words conveyed neutral concepts (e.g., the words “hometown, future, occasionally, he, recall” could be rearranged into “He occasionally recalls his hometown”). In contrast, the money concept priming group encountered money-related words in half of the sets (e.g., “is, observe, bank, high-profit, industry” could be rearranged into “Bank is a high-profit industry”), with the remaining sets containing neutral words. The scrambled-sentences task is widely used in psychology for priming or detecting cognitive biases (e.g., Vohs, 2015; Würtz et al., 2022), and its effectiveness has been systematically demonstrated in previous studies (e.g., Würtz et al., 2022).

Following the scrambled-sentences task, participants evaluated 20 moral dilemmas adapted from Conway and Gawronski (2012). These dilemmas required participants to assess whether performing a harmful action to achieve a specific outcome was morally justifiable, determining whether the described action was appropriate or inappropriate. The dilemmas included 10 basic scenarios, each with congruent and incongruent versions. In the incongruent dilemmas, the benefits of the harmful action outweighed the harm, creating a conflict between utilitarian (accepting harm) and deontological (rejecting harm) perspectives. In contrast, the congruent dilemmas minimized the benefits of causing harm, thereby aligning utilitarian and deontological responses.

Finally, participants provided demographic information, including age, gender, professional status (employed/student), monthly household income (1. Below 3000; 2. 3000–5000; 3. 5000–8000; 4. 8000–12,000; 5. 12,000–15,000; 6. 15,000–20,000; 7. 20,000–30,000; 8. Above 30,000), monthly consumption level (1. Below 1000; 2. 1000–1500; 3. 1500–2000; 4. 2000–3000; 5. 3000–4000; 6. 4000–5000; 7. 5000–8000; 8. 8000–10,000; 9. Above 10,000), and household location (urban/rural).

### 2.2. Results and Discussion

Participants deemed harmful actions acceptable in 50.10% of incongruent dilemmas and 36.27% of congruent dilemmas. There was a significant difference between the two kinds of dilemmas, *t*(101) = 9.55, *p* < 0.001.

Deontological (D parameter) and utilitarian (U parameter) scores were calculated using the formulas from Conway and Gawronski’s study (2012). A *t*-test was conducted to examine the effect of money priming on these parameters. Results showed that participants in the money concept priming group (*M* = 0.17, *SD* = 0.16) exhibited higher utilitarian inclinations compared to the control group (*M* = 0.11, *SD* = 0.13), *t*(101) = 2.28, *p* = 0.025, Cohen’s *d* = 0.45. However, there was no significant difference in D parameter (Deontological tendency) between the money concept priming group (*M* = 0.58, *SD* = 0.20) and the control group (*M* = 0.58, *SD* = 0.18), *t*(101) = 1.61, *p* = 0.87, Cohen’s *d* = 0.32.

Previous research found that demographic variables can influence individuals’ attitudes toward money and moral judgments, for instance, income level, consumer attitudes, employment status, gender, and age (Côté et al., 2013; Lu & Fung, 2019; Zaleskiewicz et al., 2018; Zulfiqar, 2023). Therefore, we also conducted an ANOVA to test the impact of money priming on U and D parameters while controlling for gender, age, occupation, household income, consumption level, and location. The analysis revealed that individuals in the money concept priming group had higher utilitarian inclinations than the control group, *F*(1, 92) = 6.32, *p* = 0.014, *η*^2^*_p_*= 0.06. In contrast, there was no significant difference between the two groups in deontological inclinations, *F*(1, 92) = 0.31, *p* = 0.58, *η*^2^*_p_* = 0.003.

The observed effect size (Cohen’s *d* = 0.45) falls within the conventional range for a small-to-medium effect in behavioral research (Cohen, 1994). To contextualize its practical relevance, this effect size suggests that the mean difference in utilitarian reasoning between the money concept priming group and the control group corresponds to approximately 67% non-overlap in distributions. This indicates a noticeable, though not overwhelming, shift in moral judgment patterns. Such an effect could have meaningful real-world implications when applied to population-level decisions. For example, in high-stakes moral dilemmas (e.g., triage scenarios, corporate ethics decisions), even a modest shift toward utilitarian preferences induced by environmental cues like money priming could systematically alter collective outcomes. Although laboratory effects may attenuate in ecological settings, this finding highlights the substantial role of situational factors in ethical cognition—a critical consideration for institutions designing choice architectures in finance, policy, or healthcare domains, where monetary cues are ubiquitous.

In summary, the concept of money significantly influenced utilitarian inclinations but did not affect deontological inclinations. Specifically, individuals primed with the concept of money exhibited stronger utilitarian tendencies compared to those primed with neutral concepts. As hypothesized, priming participants with money-related thoughts likely induced a business- and market-oriented mindset, prompting them to approach moral dilemmas through cost–benefit analysis and calculation (Liu & Aaker, 2008). This shift resulted in a heightened utilitarian orientation.

## 3. Study 2

Study 2 was designed to further investigate whether another facet of money—specifically, the “love of money”—exerts a comparable influence on moral judgments as the mere concept of money. This study focused on examining the relationship between the love of money and moral judgments, utilizing the Moral Orientation Scale (MOS) to measure four key orientations: deliberation, rule, sentiment, and integration.

Based on prior research, we hypothesized that individuals with a higher love of money would exhibit stronger utilitarian inclinations and weaker deontological tendencies in moral reasoning. This hypothesis was grounded in the expectation that the love of money would enhance deliberation orientation while diminishing rule orientation. Specifically, we anticipated that a heightened love of money would promote cognitive, effortful processing associated with increased utilitarian reasoning and reduced deontological thinking, while simultaneously reducing reliance on rule-based processing characteristic of deontological judgments.

### 3.1. Method

#### 3.1.1. Participants

G*Power was used to calculate the required sample size for the study, specifying a medium effect size (*f*^2^ = 0.15), an alpha level of 0.05, a power of 1 − *β* = 0.8, and seven predictors. The calculated sample size for the regression analysis was 103 participants. To ensure sufficient statistical power, a total of 488 participants (297 female, 191 male; age range: 18–66 years, *M* = 29.65, *SD* = 8.20) were recruited via an online platform (www.wenjuan.com) in China. The sample included 104 students (21.3%) and 384 employed individuals (78.7%), with 114 participants (23.4%) from rural areas and 374 participants (76.6%) from urban areas. All participants volunteered for the study, and none had participated in Study 1.

#### 3.1.2. Procedure and Materials

To assess participants’ attitudes toward money, we employed the Love of Money Scale (LOMS; Tang & Chiu, 2003), which consists of nine items organized into three dimensions: rich, importance, and motivation. These dimensions capture the extent to which individuals view money as a motivating factor, perceive it as important, and aspire to accumulate wealth. Participants responded to each item on a 7-point scale ranging from 1 (strongly disagree) to 7 (strongly agree). The LOMS demonstrated strong reliability (Cronbach’s α = 0.943).

Participants then completed the Moral Orientation Scale (MOS), which includes 28 items across four subscales: deliberation, rule, sentiment, and integration. These subscales assess key thinking processes involved in moral reasoning. Although our hypothesis initially focused on deliberation and rule orientations as potential mediators between the love of money and deontological/utilitarian inclinations, we included all four subscales to provide a comprehensive assessment. Participants indicated their level of agreement on a 7-point Likert scale, ranging from 1 (strongly disagree) to 7 (strongly agree). The order of items was randomized to minimize order effects. The internal consistency of the MOS subscales was satisfactory, with Cronbach’s alpha coefficients ranging from 0.73 to 0.89.

After completing the LOMS and MOS, participants made judgments on 20 moral dilemmas, identical to those used in Study 1. Finally, participants provided demographic information, including gender, age, employment status, household income, and consumption level.

### 3.2. Results and Discussion

In total, harmful actions were deemed acceptable in 53.2% (*SD* = 0.19) of incongruent dilemmas and 38.0% (*SD* = 0.18) of congruent dilemmas. There was a significant difference between these two types of dilemmas, t(487) = 18.09, *p* < 0.001.

Deontological (D parameter) and utilitarian (U parameter) scores were calculated using the formulas from Conway and Gawronski’s study (2012). Regression analysis was conducted to test the relationship between the love of money and D and U parameters. Results showed that the love of money positively predicted utilitarian thinking, *β* = 0.01, *t*(486) = 2.17, *p* = 0.03, 95% CI [0.001, 0.03], and negatively predicted deontological inclinations, *β* = −0.21, *t*(486) = −4.62, *p* < 0.001, 95% CI [−0.05, −0.02].

Similar to Study 1, we also tested the relationship between love of money and deontological/utilitarian inclinations with gender, age, occupation, household income, consumption level, and location of household as control variables. It was found that love of money was positively associated with individuals’ utilitarian inclinations and negatively related to individuals’ deontological inclinations (see Table 1).

Higher levels of love of money positively predicted deliberation orientation, *β* = 0.22, *t*(486) = 5.00, *p* < 0.001, 95% CI [0.08, 0.19], and rule orientation, *β* = 0.10, *t*(486) = 2.18, *p* = 0.03, 95% CI [0.008, 0.15]. However, love of money was not related to sentiment, *β* = 0.03, *t*(486) = 0.56, *p* = 0.57, 95% CI [−0.06, 0.10], and integration orientations, *β* = −0.03, *t*(486) = −0.67, *p* = 0.50, 95% CI [−0.09, 0.04].

**Figure 1:**
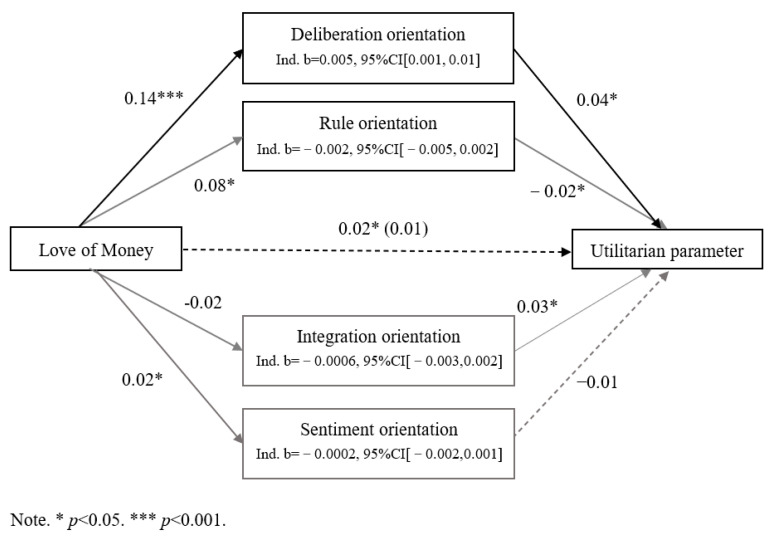
Mediation model for the effect of love of money on utilitarian parameters.

We employed the Process macro for SPSS 26 (Model 4; Hayes, 2013) to investigate whether any of the four moral orientations mediated the impact of the love of money on utilitarian inclinations, using 5000 bootstrapped samples. The results indicated that only the deliberation orientation mediated this effect (see Figure 1). Specifically, the love of money was associated with the utilitarian parameter positively, *b* = 0.016, *SE* = 0.007, *t*(486) = 2.17, *p* = 0.03, 95% CI [0.002, 0.03]. Additionally, the love of money positively predicted deliberation, *b* = 0.14, *SE* = 0.03, *t*(486) = 5.00, *p* < 0.001, 95% CI [0.08, 0.19]. Deliberative thinking, in turn, was positively associated with U parameter, with *b* = 0.04, *SE* = 0.02, *t*(482) = 2.46, *p* = 0.014, and a 95% CI of [0.007, 0.07]. We also identified an indirect effect on the U parameter, with *b* = 0.005, *SE* = 0.003, and a 95% CI of [0.001, 0.01].

When all four mediating variables were included in the model, the influence of the love of money on U parameter diminished, with *b* = 0.13, *SE* = 0.007, *t*(482) = 1.81, *p* = 0.071, and a 95% CI of [−0.001, 0.03]. This suggests that the love of money is associated with an increase in utilitarian inclinations primarily because it enhances deliberation orientation.

**Figure 2:**
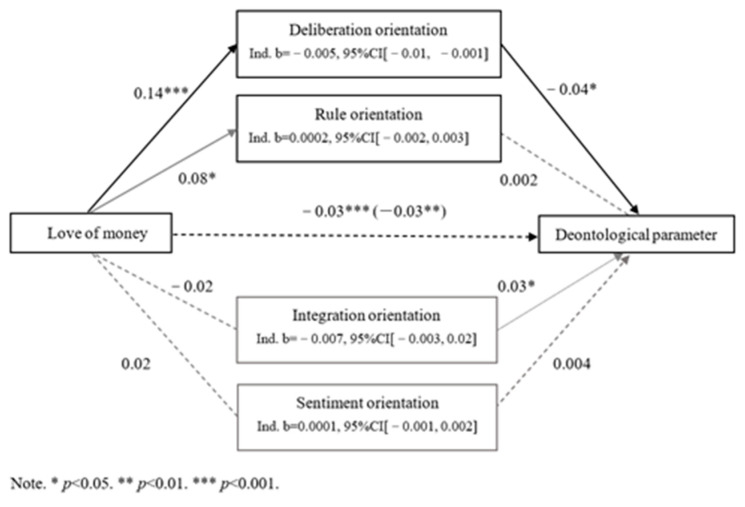
Mediation model for the effect of love of money on deontological parameters.

Additionally, we assessed whether any of the four moral orientations mediated the impact of the love of money on deontological inclinations using 5000 bootstrapped samples. The results showed that only the deliberation orientation partially mediated this effect (see Figure 2). Specifically, the love of money is negatively related to D parameter, with a coefficient of *b* = −0.03, standard error (SE) of 0.007, *t*(486) = −4.62, *p* < 0.001, and a 95% confidence interval (CI) of [−0.05, −0.02]. Meanwhile, the love of money positively predicted deliberation, with *b* = 0.14, *SE* = 0.027, *t*(486) = 5.00, *p* < 0.001, and a 95% CI of [0.08, 0.19]. Deliberation, in turn, was negatively related to D parameter, with *b* = −0.04, *SE* = 0.015, *t*(482) = −2.56, *p* = 0.01, and a 95% CI of [−0.07, −0.009]. We also identified an indirect effect on the D parameter, with *b* = −0.005, *SE* = 0.003, and a 95% CI of [−0.01, −0.001]. After including all four mediating variables, the influence of love of money on D parameter was reduced, with *b* = −0.03, *SE* = 0.008, *t*(482) = −3.68, *p* = 0.0003, and a 95% CI of [−0.069, −0.009]. This indicates that deliberation partially mediated the impact of the love of money on deontological inclinations, suggesting that an increased love of money is associated with a decrease in deontological inclinations primarily through an increase in deliberation orientation.

Study 2 focused on another salient aspect of money: love of money. Specifically, it explored how individuals’ attitudes toward money influence moral judgments. Similar to the priming of money concepts in Study 1, Study 2 revealed that the love of money is positively associated with utilitarian inclinations. However, in contrast to the effect of money concept priming, the love of money was found to be negatively related to deontological inclinations.

As outlined in the introduction, priming the concept of money and possessing a desire for money carry distinct connotations, and thus, their impacts on individual cognition and behavior are not identical. Priming the concept of money activates a mindset more oriented toward calculation. In contrast, a long-term love of money may not only limit individuals to a focus on benefits and costs but also promote indifference toward others and a disregard for fundamental moral norms. Simultaneously, it may foster a greater concern for the collective interests of the group. In this way, the love of money leads to stronger utilitarian inclinations and weaker deontological inclinations.

Additionally, Study 2 investigated the underlying mechanisms driving these effects, discovering that the love of money influences both deontological and utilitarian inclinations by enhancing deliberation orientation. This suggests that the love of money may amplify cognitive and effortful processing associated with utilitarian reasoning, while simultaneously reducing reliance on the intuitive processing characteristic of deontological judgments.

Contrary to our hypothesis, the rule orientation did not mediate the relationship between the love of money and deontological inclinations. We propose that the rule orientation scale may not fully capture the essence of how the love of money diminishes deontological thinking. This may be because the underlying mechanism involves not only a disregard for general rules but, more significantly, a reduced focus on fundamental moral principles such as care for others and the prohibition against harm.

## 4. General Discussion

Although prior research has examined the impact of money on moral-related variables such as unethical, self-serving, and prosocial behaviors (e.g., Enke, 2023; Gino & Mogilner, 2014; Kouchaki et al., 2013), empirical studies specifically investigating how money influences moral judgments remain sparse. The present research explores the impact of money—both the concept of money and the love of money—on deontological and utilitarian moral thinking in moral judgments using the process-dissociation technique. We hypothesized that priming the concept of money would increase utilitarian moral inclinations, and that the long-term trait of loving money would reduce deontological inclinations while enhancing utilitarian inclinations. Additionally, we examined whether moral orientations mediate the influence of the love of money on deontological and utilitarian tendencies.

Our findings provide support for these hypotheses. In Study 1, we explored the impact of money on moral judgments by priming participants with money-related concepts. Results indicated that those primed with money concepts exhibited a greater tendency toward utilitarianism in their moral judgments compared to participants primed with neutral concepts, with no significant differences observed in deontological thinking between the two conditions. Study 2 examined the effect of long-term attitudes toward money, specifically focusing on the love of money. We found that individuals with a higher love of money had lower deontological inclinations and higher utilitarian inclinations. This suggests that the love of money has a distinct influence on moral reasoning compared to the mere activation of money-related concepts. Moreover, Study 2 identified a mediating role of deliberation orientation in the relationship between the love of money and moral judgments.

Our observation that both priming the concept of money and the love of money enhance utilitarian reasoning aligns with previous research on market mindset activation (Zaleskiewicz et al., 2020). Their research found that priming a market mindset increases people’s tendency to make utilitarian moral choices through proportional thinking, a defining feature of the market mindset. Our study extends these findings by showing that priming the concept of money also increases utilitarian moral thinking, suggesting that money-related priming induces a cognitive style similar to that activated by a market mindset. Moreover, prior research has shown that money fosters a tendency to calculate benefits and costs and to maximize personal gains (Beus & Whitman, 2017; Vohs, 2015). This market mindset increases the inclination to make utilitarian moral choices. Our findings are consistent with these studies, showing that both priming the concept of money and an individual’s love of money lead to a mindset focused on cost–benefit analysis and market-oriented thinking.

Additionally, previous research has found a positive correlation between money and prosocial behavior, as well as cooperation (Enke, 2023; Henrich et al., 2010). The positive association between the love of money and utilitarian tendencies is also consistent with these findings. Specifically, individuals with a monetary mindset tend to approach moral dilemmas from the perspective of collective welfare rather than personal gain.

The finding that the love of money reduces deontological thinking aligns with a body of research showing that money can lead to unethical behaviors, such as focusing on personal achievement (Beus & Whitman, 2017), reduced prosocial actions (Gasiorowska et al., 2016; Mead & Stuppy, 2014), diminished empathy (Kraus et al., 2010; Ma-Kellams & Blascovich, 2013), and a tendency to treat others as instruments for self-gain (Wang & Krumhuber, 2017). For example, a study examined how money impacts empathy (Ma-Kellams & Blascovich, 2013), finding that participants who received a financial incentive for performing empathic accuracy tasks displayed less empathy. Similarly, Study 2 found that the love of money made individuals more likely to harm a few to benefit many, indicating a reduction in deontological inclinations.

Nevertheless, this finding appears to be inconsistent with anthropological text analyses that document positive associations between money, markets, and prosociality, moral values, and fairness (Enke, 2023; Henrich et al., 2010). Given that deontological reasoning primarily reflects adherence to established moral principles (e.g., prohibitions against harm), the connection between money and moral values would be expected to enhance—rather than diminish—deontological inclinations. While Enke (2023) and Henrich et al. (2010) observed associations between money and generalized moral values (e.g., fairness, cooperation), their operationalization of morality does not specifically address the deontological–utilitarian dichotomy central to our framework. This lack of clarity prevents direct comparisons: Their measures of “prosociality”, “moral values”, and “moral emotion” could correspond either to (a) deontological adherence to harm-avoidance norms or (b) utilitarian optimization of collective welfare. Without clear theoretical alignment with these distinct moral frameworks, their findings cannot resolve whether market-integrated societies prioritize rule-based ethics or consequence-driven ethics. Furthermore, we argue that this divergence arises from fundamental methodological differences: While previous textual analyses employed a macro-level perspective to identify cultural narratives linking money to collective ethics, our study focused on individual-level psychological processes—specifically, how trait-level monetary desire (love of money) interacts with moral cognition.

The effect of money on decreased deontological thinking was observed only in individuals with a high love of money, not in those primed with money-related concepts. This supports our hypothesis that priming the concept of money and individuals’ love for money exert differential influences on cognition and behavior. Specifically, priming money-related thoughts induces a market-oriented mindset characterized by a focus on cost–benefit analyses and economic rationality (Bijleveld & Aarts, 2014; Liu & Aaker, 2008). In contrast, a long-term love of money has a more profound impact on individuals’ values, cognitive processes, and behaviors. This sustained market-oriented mindset may diminish empathy, foster a disregard for moral principles, and increase selfish tendencies (Gasiorowska et al., 2016; Kraus et al., 2010; Ma-Kellams & Blascovich, 2013). Thus, the love of money is associated with stronger utilitarian inclinations and weaker deontological tendencies. Furthermore, the love of money may prompt individuals to consider societal welfare, fostering a more positive form of utilitarianism that prioritizes collective good over individual gain (Enke, 2023; Henrich et al., 2010).

Regarding the mediating effects, our hypotheses were partially supported. The study identified a mediating role of deliberation orientation in the relationship between the love of money and both utilitarian and deontological inclinations. This suggests that the love of money encourages more deliberate calculations of gains and losses. However, deliberation orientation only partially accounted for the effect of the love of money on deontological thinking. This indicates that additional mechanisms beyond deliberation may explain the relationship between the love of money and deontological inclinations, and future research should explore these further.

Our findings on the mediating role of deliberative orientation differ somewhat from previous studies. For instance, Zaleskiewicz et al. (2020) attributed utilitarian shifts to proportional thinking, while our research emphasizes cognitive effort as the primary driver, positioning our work within Greene’s (2009) dual-process model, where deliberation mediates the utilitarian override of intuitive deontology.

Meanwhile, no mediating effect of rule orientation was found. This may be attributed to the fact that the rule orientation scale primarily measures adherence to general social rules. In contrast, the moral dilemmas presented in our study largely focused on moral principles related to care and harm toward others. Consequently, a general measure of rule orientation may not adequately capture participants’ attitudes toward these specific moral principles. As a result, no mediating effect of rule thinking in the relationship between the love of money and deontological inclinations was observed. Future research might consider utilizing more specialized measures that focus on attitudes toward moral principles of care and harm.

In classical sociological works by scholars such as Marx (1976), Weber (1978), and Simmel (1907/2004), money is conceptualized as a catalyst for individualism, prompting individuals to adopt a calculative perspective when engaging with the world and addressing problems. This orientation is centered on personal gains and losses, often at the expense of social connections and the devaluation of fundamental moral principles. In contrast, anthropological research has highlighted the interplay between monetary systems, market exchanges, and prosocial moral concepts such as cooperation and trust. These studies suggest that money and markets can foster a mindset oriented toward collaborative endeavors (Enke, 2023; Henrich et al., 2010). These seemingly divergent perspectives are not mutually exclusive.

Money, as a multifaceted concept, exerts a nuanced and varied influence on both societal structures and individual behaviors. Consistent with Lea and Webley’s (2006, 2014) dual-process framework, money embodies both instrumental utility as a transactional tool and maladaptive salience through its compulsive, drug-like qualities—a dichotomy that reflects its capacity to rationally mediate exchanges while paradoxically hijacking reward systems to perpetuate non-functional incentives.

The current research extends Marx’s and Simmel’s critiques into the cognitive domain: Just as money alienates labor under capitalism (Marx, 1976), it also alienates moral reasoning from relational imperatives when internalized as a trait. As a situational tool, however, money’s utilitarian bias may reflect adaptive market logic (Henrich et al., 2010)—a duality that can be reconciled through Lea and Webley’s (2006, 2014) tool-drug framework.

Building on prior empirical research, we propose that the love of money may simultaneously foster both negative utilitarianism (where individuals become more self-interested and indifferent to others) and positive utilitarianism (a mindset oriented toward cooperation and trust). Our current data do not conclusively support this hypothesis, as they indicate that individuals with a higher love of money engage more in deliberative thinking, leading to stronger utilitarian inclinations and weaker deontological inclinations. It is possible that the moral orientation scale used in this study, serving as a mediating variable, did not sufficiently capture finer-grained moral attitudes, such as empathy for others, endorsement of harm, or prioritization of collective and societal welfare. Future studies could specifically examine the roles of these orientations.

Overall, our investigation into the impact of money on moral judgments advances the understanding of how money influences individual cognition and behavior. The manner in which money affects moral thinking is consistent with prior research on money’s effects on unethical behavior, prosocial behavior, and other moral variables. Furthermore, the distinct influences of priming the concept of money and the long-term trait of loving money on moral reasoning corroborate various theories of money. On the one hand, money can have instrumental effects, such as activating a calculative mindset when individuals are primed with money-related concepts. On the other hand, it can also act in ways analogous to the drug theory perspective, fostering indifference toward others and diminishing deontological inclinations. Additionally, our finding that the love of money influences moral judgments by increasing deliberation orientation aligns with the dual-process model of moral judgment, thereby lending support to this theoretical framework.

The findings also carry practical implications. Both temporary thinking about money and long-term love of money promote a focus on cost–benefit analysis and a market-oriented mindset. Meanwhile, the love of money may lead individuals to prioritize self-interest and disregard moral rules. At a societal level, widespread love of money could contribute to more self-serving and unethical behaviors. If systemic issues in society fuel this mindset, it could become a pervasive social problem. Governments should consider addressing this issue through policies and societal measures that encourage individuals to focus on concerns beyond economics, such as addressing economic instability and social inequality. Promoting attention to fundamental moral issues—such as caring for and helping others—could foster ethical behavior in society, beyond mere monetary and economic interests.

### 4.1. Process-Dissociation Procedure

In the current research, we utilized the Process-Dissociation (PD) procedure to investigate participants’ moral judgments. The PD procedure offers several advantages, particularly its ability to effectively differentiate between deontological and utilitarian inclinations. This distinction helps avoid the conflation of these two orientations, which is a limitation in traditional moral dilemma paradigms where they are treated as opposing ends of a single continuum. However, the use of the PD procedure in studying moral judgments has been subject to critique by several researchers (Baron & Goodwin, 2020; Gawronski et al., 2017; Kunnari et al., 2020).

Kunnari et al. (2020) pointed out that significant variation in the content of moral dilemmas used in the PD procedure leads to low correlations between different dilemmas. Furthermore, they observed that both the utilitarian (U) and deontological (D) parameters were highly correlated with traditional dilemma scores. While the correlation between U and D parameters was found to be zero, a discernible pattern emerged: Smaller U parameters were associated with D parameters clustering around 0.5, while larger U parameters correlated with more extreme D parameters. Since U and D parameters in the PD procedure are derived from mathematical formulas, Kunnari et al. suggested that some correlations between PD parameters might be mathematical artifacts. They also argued that the lack of correlation between U and D parameters hinges on individual variations in responses to congruent dilemmas, referring to what Baron and Goodwin (2020) termed “perverse responses”, which involve accepting harm that violates norms and cannot be justified by its consequences.

However, we contend that the inclusion of diverse types and intensities of moral dilemmas in the PD procedure effectively reflects the complexity and diversity of moral judgments, thereby enhancing the generalizability of the research. As long as the congruent and incongruent dilemmas in each context exhibit consistent tendencies, they can be meaningfully compared, even without complete consistency. Moreover, the correlation between U and D parameters and traditional dilemmas does not indicate a flaw in the paradigm, as these parameters are calculated using mathematical formulas based on traditional moral dilemmas. The observed pattern in the relationship between U and D parameters does not undermine the paradigm’s capacity to successfully distinguish between the two coefficients.

Regarding the issue of perverse responses, Gawronski et al. (2020) argued that this criticism implicitly assumes that experimental manipulations are valid only when judgments are driven by conscious deliberation about moral theories. However, this perspective conflates moral choices with explanatory psychological structures and overlooks an important theory in moral research: Moral judgments are not necessarily the result of conscious deliberation about moral theories (Greene et al., 2008; Haidt, 2001). For instance, Greene et al.’s (2008) dual-process theory of moral judgment suggested that judgments aligned with moral norms are often driven by emotional processes, without involving conscious deliberation about those norms.

While we acknowledge that the PD procedure has limitations, we believe it remains an effective tool for investigating moral judgments. Some scholars have noted additional limitations of the PD procedure. Although the PD model recognizes the conceptual and empirical distinction between utilitarian and deontological inclinations, it conflates these inclinations with a general preference for inaction versus action. Specifically, the U parameter combines sensitivity to consequences with a preference for action, and the D parameter combines sensitivity to moral norms with a preference for inaction. In response to this, scholars have proposed alternative models, such as the CNI model (Gawronski et al., 2017) and the CAN algorithm (Liu & Liao, 2021), to more precisely capture the cognitive processes involved in moral judgments. To further validate the findings of the present study, future research could consider using these models to examine the impact of money-related thinking on moral judgments.

### 4.2. Limitations and Future Directions

The present study has several limitations. First, participants were recruited through an online platform, with a relatively broad age range (18–45 years in Study 1 and 18–66 years in Study 2). However, the average age of participants was relatively young (Study 1: *M* = 22.53, *SD* = 5.72; Study 2: *M* = 29.65, *SD* = 8.20), and all participants were from China. As such, it remains unclear whether the findings of this study can be generalized to other cultural contexts. To address these limitations, future research could employ a cross-cultural design with stratified sampling, recruiting age-matched subgroups of older adults and participants from non-Chinese cultural backgrounds, thereby disentangling potential age and cultural confounds.

Additionally, while we measured participants’ employment status, income levels, consumption levels, and residential areas as indicators of socioeconomic status, more detailed profiling could offer further insights. For instance, longitudinal tracking of participants’ financial hardship history or examining occupation-specific monetary salience (e.g., comparing finance professionals to artists) could reveal subgroup susceptibilities to money priming that may have been obscured in our aggregated analyses.

In Study 1, money priming was achieved using a scrambled-sentences task. To mitigate potential demand characteristics associated with explicit semantic activation tasks, post hoc manipulation checks were intentionally omitted, consistent with implicit priming methodologies (Bargh, 1994). While this design preserved the ecological validity of unconscious mental state engagement, it precluded direct psychometric verification of the priming mechanism’s efficacy—a trade-off often seen in covert experimental manipulations. Future research could incorporate indirect validity measures, such as embedding implicit checks (e.g., post-experimental word-completion tests assessing spontaneous money-related associations) or tracking psychophysiological markers (e.g., pupil dilation during priming) to verify subconscious processing of monetary cues without alerting participants to the manipulation’s purpose.

We also acknowledge that the moral dilemmas used in our study, such as scenarios involving the suffocation of a baby to save a village or permitting a daughter to engage in pornographic activities for economic survival, may be seen as extreme and not fully representative of the moral challenges individuals face in everyday life. Therefore, future research should consider using more relatable moral scenarios to validate the findings. For example, dilemmas involving workplace ethics, environmental decisions, or community interactions could provide a more realistic context for examining the influence of money on moral reasoning.

We recognize that alternative interpretations of the findings are possible. In Study 1, the observed increase in utilitarian thinking inclinations following money priming via scrambled-sentence tasks might reflect heightened cognitive effort rather than a direct effect of monetary cognition. Specifically, the inclusion of money-related terms (e.g., “profit”, “bank”) in the priming condition may have unintentionally increased task complexity compared to neutral sentences, necessitating more deliberate processing—a cognitive state known to amplify utilitarian preferences (Greene et al., 2008). To address this potential confound, future research should employ priming paradigms that separate monetary salience from cognitive load, such as behavioral economic tasks (Zaleskiewicz et al., 2020), autobiographical recall paradigms (Vohs, 2015), or visual priming methods (Zhou et al., 2009).

In Study 2, we found that the love of money was positively correlated with utilitarian inclinations and negatively correlated with deontological tendencies. However, this relationship may be confounded by unmeasured variables such as financial scarcity (i.e., chronic lack of monetary resources) and power dynamics—constructs that are conceptually adjacent to, but distinct from, monetary desire. For instance, individuals experiencing financial precarity may develop heightened monetary fixation and adopt utilitarian heuristics as adaptive survival strategies, while affluent individuals may associate money with agency rather than scarcity (Sharma & Alter, 2012). To disentangle these effects, future studies should control for financial scarcity and sense of power. Measures such as the Material Deprivation Index (MDI) could be used to isolate trait-level monetary desire from situational economic constraints, and further research could also assess participants’ sense of power to test whether money’s moral effects are mediated by perceived power.

## 5. Conclusions

The present study examined the relationship between money and moral judgments. Our findings suggest that the concept of money increases individuals’ utilitarian inclinations in moral decision-making, while not influencing deontological inclinations. Participants primed with money-related concepts exhibited higher utilitarian inclinations compared to those primed with neutral concepts. Moreover, the love of money was negatively associated with deontological inclinations and positively associated with utilitarian inclinations, due to its effect on increasing individuals’ deliberation orientation.

## Figures and Tables

**Table 1:** The effects of love of money on deontological (D) and utilitarian (U) inclinations.

	*D*				*U*			
	*β*	*t*	*p*	95% CI	*β*	*t*	*P*	95% CI
Love of money	−0.20	−4.50	<0.001	[−0.05, −0.02]	0.11	2.42	0.02	[0.003, 0.03]
Gender	0.08	1.70	0.09	[−0.005, 0.06]	0.01	0.18	0.86	[−0.03, 0.04]
Age	−0.04	−0.73	0.47	[−0.003, 0.001]	0.06	1.25	0.21	[−0.001, 0.004]
Occupation	−0.02	−0.34	0.73	[−0.06, 0.04]	0.09	1.53	0.13	[−0.01, 0.09]
Household location	−0.04	−0.74	0.46	[−0.06, 0.03]	0.03	0.73	0.47	[−0.03, 0.06]
Household income level	0.13	2.15	0.03	[0.001, 0.03]	0.06	0.94	0.35	[−0.007, 0.02]
Consumption level	−0.05	−0.73	0.47	[−0.02, 0.008]	−0.001	−0.009	0.99	[−0.01, 0.01]
*F*	4.34 ***				2.68 *			
*R* ^2^	0.06				0.04			
Modified *R*^2^	0.05				0.02			

Note. * *p* < 0.05; *** *p* < 0.001.

## Data Availability

The datasets used and/or analyzed during the current study are available from the corresponding author on reasonable request.
